# Early mobilisation in intensive care units in Australia and Scotland: a prospective, observational cohort study examining mobilisation practises and barriers

**DOI:** 10.1186/s13054-015-1033-3

**Published:** 2015-09-14

**Authors:** Meg E. Harrold, Lisa G. Salisbury, Steve A. Webb, Garry T. Allison, on behalf of the Australia and Scotland ICU Physiotherapy Collaboration

**Affiliations:** School of Physiotherapy and Exercise Science, Faculty of Health Science, Curtin University, GPO Box U1987, Perth, 6845 Australia; Intensive Care Unit and Physiotherapy Department, Royal Perth Hospital, Perth, Australia; School of Health in Social Science, University of Edinburgh, Edinburgh, UK; Department of Medicine and Pharmacology, University of Western Australia, Perth, Australia

## Abstract

**Introduction:**

Mobilisation of patients in the intensive care unit (ICU) is an area of growing research. Currently, there is little data on baseline mobilisation practises and the barriers to them for patients of all admission diagnoses.

**Methods:**

The objectives of the study were to (1) quantify and benchmark baseline levels of mobilisation in Australian and Scottish ICUs, (2) compare mobilisation practises between Australian and Scottish ICUs and (3) identify barriers to mobilisation in Australian and Scottish ICUs. We conducted a prospective, observational, cohort study with a 4-week inception period. Patients were censored for follow-up upon ICU discharge or after 28 days, whichever occurred first. Patients were included if they were >18 years of age, admitted to an ICU and received mechanical ventilation in the ICU.

**Results:**

Ten tertiary ICUs in Australia and nine in Scotland participated in the study. The Australian cohort had a large proportion of patients admitted for cardiothoracic surgery (43.3 %), whereas the Scottish cohort had none. Therefore, comparison analysis was done after exclusion of patients admitted for cardiothoracic surgery. In total, 60.2 % of the 347 patients across 10 Australian ICUs and 40.1 % of the 167 patients across 9 Scottish ICUs mobilised during their ICU stay (*p* < 0.001). Patients in the Australian cohort were more likely to mobilise than patients in the Scottish cohort (hazard ratio 1.83, 95 % confidence interval 1.38–2.42). However, the percentage of episodes of mobilisation where patients were receiving mechanical ventilation was higher in the Scottish cohort (41.1 % vs 16.3 %, *p* < 0.001). Sedation was the most commonly reported barrier to mobilisation in both the Australian and Scottish cohorts. Physiological instability and the presence of an endotracheal tube were also frequently reported barriers.

**Conclusions:**

This is the first study to benchmark baseline practise of early mobilisation internationally, and it demonstrates variation in early mobilisation practises between Australia and Scotland.

## Introduction

Intensive care units (ICUs) provide patients with support for failing organs during acute illness. Owing to the enforced bed rest associated with these treatments, patients often experience muscle wasting even within a few days of inactivity. Some patients develop critical illness neuropathies and myopathies that result in levels of weakness beyond that of bed rest alone [1–8]. Although not fully understood, it is thought that this immobility in conjunction with systematic inflammation may result in the muscle wasting observed [7, 9–11]. These levels of weakness are associated with longer ventilation and ICU stay [12]. This muscle weakness affects physical ability and activities of daily living beyond intensive care. Some studies have demonstrated that this weakness may continue months and even years after discharge from intensive care [13–17].

Mobilisation and exercise have been shown to produce improvements in muscle strength and respiratory function and reduction in cardiovascular complications in chronic obstructive pulmonary disease and heart failure patient populations [18, 19]. There is a growing body of evidence that early mobilisation as an intervention in intensive care may improve recovery [20–22]. Several studies have demonstrated that early mobilisation in specific subgroups of patients in ICU is feasible and safe [23–27]. However, the utility of early mobilisation on patient-centred outcomes is unclear. The literature on mobilisation in ICU is dominated by cohorts of patients admitted with respiratory failure. In many level 3 ICUs, this is not a leading cause of admission [28]. Furthermore, it is difficult to generalise data if mobilisation strategies are introduced into ICUs in the absence of any benchmarking of practise or barriers to mobilisation. In addition, it is unknown how practise varies between different ICUs or countries. To date, there are no studies that document the barriers and delivery of mobilisation in patients of all admission diagnoses throughout their entire length of stay at multiple sites and countries recorded in real time.

The purpose of this study was to evaluate baseline practise and the perceived barriers to early mobilisation in ICU across multiple sites in two different countries with different systems of health care delivery. We examined Australian and Scottish ICU patient populations and report the details of type, number and duration of mobilisation activities as well as the perceived barriers to conducting mobilisation recorded at the bedside.

### Objectives

The following were the aims of this study:

1. To quantify and benchmark levels of mobilisation in Australian and Scottish ICUs in all patients who received mechanical ventilation
2. To compare mobilisation practises between Australian and Scottish ICUs
3. To identify barriers to mobilisation in Australian and Scottish ICUs

## Methods

### Design

A prospective observational cohort study was conducted in 10 ICUs in Australia and 9 ICUs in Scotland. Recruitment occurred during a 4-week inception period, and patients were censored for follow-up at ICU discharge or after 28 days (4 weeks), whichever occurred first. The follow-up period of up to 28 days was chosen on the basis of data that indicated that >97 % of all patients admitted to ICUs in Australia are discharged within 25 days (Royal Perth Hospital quality assurance database, 2007–2009).

### Setting

#### Locations and recruitment

Sites were included in the study if they had established administrative databases that collected demographic and baseline clinical information and the site physiotherapist investigator was able to access this information. All sites were tertiary metropolitan ICUs with level 3 ICU beds.

### Participants

#### Inclusion criteria

Patients aged >18 years, admitted to a participating ICU and receiving mechanical ventilation in the ICU were eligible.

#### Exclusion criteria

There were no exclusion criteria.

### Research process

Mobilisation activities were defined as those in which movement was against gravity and involved axial loading of the spine and/or long bones. These activities, in hierarchical order of difficulty from lowest to highest were (1) sitting over the edge of the bed, (2) sitting in a chair, (3) use of a tilt table to ≥40 degrees and (4) standing and ambulating. Physical and/or mechanical assistance was permitted to complete these activities.

An episode of mobilisation was defined as a single continuous period of mobilisation with a period of bed rest on either side of that session. An episode of mobilisation could include more than one activity. If more than one episode of mobilisation occurred per day, this was recorded.

All patient and mobilisation details were recorded by physiotherapists working in the unit using a standardised mobilisation data collection form that was developed and piloted before the study [29]. Activities and episodes of mobilisation were collected for all newly admitted patients who met the inclusion criteria for their entire length of stay in ICU. This included events that occurred whilst intubated and extubated throughout the patient’s stay. Activities that occurred outside physiotherapy treatments were recorded by nursing staff on observation charts and then transcribed by the physiotherapist onto the data collection form.

Perceived barriers to mobilisation were collected on the same form by the treating physiotherapist, from a list of predefined and non-predefined barriers. Predefined barriers included patient sedated, comatose, procedure required, endotracheal tube (ETT) in situ, renal replacement therapy in progress, lack of resources, patient refusal, orthopaedic orders, imminent death, diarrhoea, cardiovascular system (CVS) unstable, central nervous system (CNS) unstable and respiratory system unstable. Non-predefined barriers were recorded individually under the ‘other’ category. Barriers were recorded only if a patient was seen by a physiotherapist and an episode of mobilisation did not occur. More than one barrier could be recorded. Barriers were recorded at the time of decision making at the bedside.

Descriptive data on unit size and resources available were obtained via a questionnaire completed by the most senior physiotherapist working at each site.

#### Sample size expectations

The sample size of the number of ICUs recruited was one of convenience.

### Statistical analysis

IBM SPSS Statistics version 21 software (IBM, Armonk, NY, USA) was used for analysis. All data were assessed for normality. Parametric data are reported as mean and standard deviation (SD), and between-groups analyses were conducted using two-sided independent *t* tests. Non-parametric categorical data are reported using frequency and percentage and were analysed using χ^2^ tests. Non-parametric interval data are reported as median and interquartile range (IQR), and between-groups analysis was done using the Mann–Whitney *U* test. Statistical significance was indicated by a *P* value <0.05.

### Ethical approval

This study was observational in nature and did not pose substantial ethical risk. Ethical approval was obtained for all 10 Australian hospitals with a waiver of consent (see Acknowledgements). A waiver of ethical approval was granted for Scotland, and Caldicott guardianship was required and obtained for each National Health Service (NHS) region (see Acknowledgements).

## Results

### Patient and ICU characteristics

Data were collected in 10 ICUs in Australia (659 patients) and nine ICUs in Scotland (171 patients) between November 2010 and December 2011. The average number of beds in Australian ICUs was 19.4 (range 10–34, SD 7.2), which was significantly greater than the Scottish ICUs (8.3, range 4–19, SD 3.3; *p* = 0.014). Staffing levels varied between sites. However, the mean ratio of physiotherapists to ICU beds for the Australian cohort was 1:5.6 (min 1:3.0, max 1:8.5, SD 1.8), and for the Scottish cohort it was 1:6.7 (min 1:3.3, max 1:10, SD 2.4), which was not statistically different (*p* = 0.298).

The characteristics of patients in the Australian and Scottish cohorts are reported in Table 1. No Scottish site admitted patients after cardiothoracic surgery, whereas 43.3 % of all patients in the Australian cohort were admitted after cardiothoracic surgery. For this reason, patients admitted for cardiothoracic surgery were identified and compared with the remaining Australian cohort (Table 2). Significant differences were noted in severity of illness and length of stay in both ICU and hospital. Given these differences and the lack of a comparative group in the Scottish cohort, analysis was then undertaken between the Australian cohort, excluding cardiothoracic patients and patients with a missing diagnosis, and the Scottish cohort, excluding patients with a missing diagnosis.

**Table 1:** Baseline demographic results for Australian and Scottish cohorts

		Australia	Scotland	*p*-value
Number of patients		659 (100 %)	171 (100 %)	
Admission diagnosis				
	Medical	169 (25.6 %)	97 (56.7 %)	0.072
	Surgical	427 (286 CTx; 43.3 %) (64.8 %)	60 (0 CTx) (35.1 %)	
	Trauma	37 (5.6 %)	10 (5.8 %)	
	Missing	26 (3.9 %)	4 (2.3 %)	

*Abbreviation*: *CTx* cardiothoracic surgery

Admission diagnosis assigned using Acute Physiology and Chronic Health Evaluation III diagnostic categories

**Table 2:** Demographic details for Australian cohort with cardiothoracic surgery diagnosis and other diagnoses and Scottish cohort

	Australia		Scotland
Diagnostic group	Cardiothoracic surgery	All other	All other
Number of patients	286	347	167
Sex (% male)	72.7^a^	61.1	58.2
Age, yr, mean (SD)	64.7^a^	55.9 (18.1)	55.6 (17.0)
APACHE II score, mean (SD)	14.8^a^	18.4 (8.3)	18.2 (7.2)
LOS ICU, days median (IQR)	1.1 (0.92–2.2)^a^	3.0 (1.3–6.1)	3.7 (1.9–7.9)^b^
LOS hospital, days, median (IQR)	9.9 (6.9–14.9)^a^	12.8 (6.5–22.0)	15.0 (6.0–29.0)

*Abbreviations*: *APACHE II* Acute Physiology and Chronic Health Evaluation II; *ICU* intensive care unit, *IQR* interquartile range, *LOS* length of stay, *SD* standard deviation

^a^Statistically significant difference between the Australia Cardiothoracic surgery and Australia All other category

^b^Statistically significant difference between the Australia All other category and the Scotland All other category

### Mobilisation

**Fig. 1:**
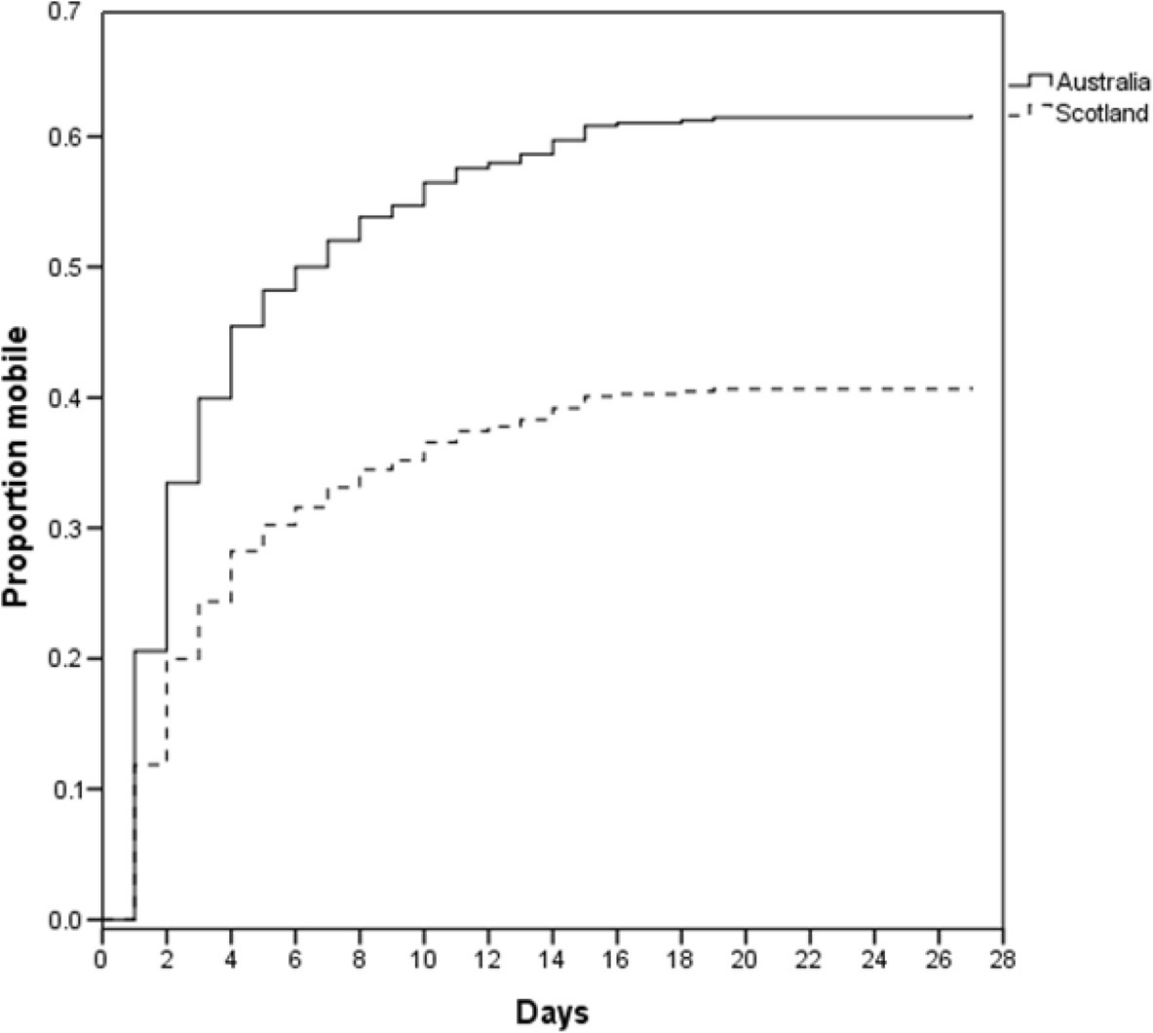
Time to mobilisation for Australian and Scottish cohorts, excluding cardiothoracic surgery patients

Mobilisation results varied between Australia and Scotland (see Table 3). A greater proportion of ICU patients were mobilised in Australia (209 [60.2 %] of 347) than in Scotland (68 [40.1 %] of 167) (*p* < 0.001). The proportion of patients mobilised and time to mobilisation was plotted using Kaplan-Meier survival curves with adjustment for Acute Physiology and Chronic Health Evaluation II score (see Fig. 1). Patients in the Australian cohort had a higher chance of earlier mobility than patients in the Scottish cohort (hazard ratio 1.83, 95 % confidence interval 1.38–2.42). The number of episodes of mobilisation undertaken per mobilised patient per ICU day was not different between the Australian cohort and the Scottish cohort (0.65 [0.35– 1.04] vs 0.44 [0.21–1.35]; *p* = 0.322). Similarly, the median number of activities conducted per patient per ICU day was also similar between the cohorts (1.0 [0.37–1.77] vs 0.69 [0.30–3.00]; *p* = 0.882). The average duration of each mobilisation episode was significantly higher in the Scottish cohort (median 157 minutes [IQR 103–239 minutes]) than in the Australian cohort (median 105 minutes [IQR 14–191 minutes]) (*p* < 0.001).

**Table 3:** Mobilisation results for the Australian and Scottish cohorts

	Australia	Scotland	*p* value
Percentage of patients who mobilised	60.2 % (209/347)	40.1 % (68/167)	<0.001^a^
Number of activities (total)	870	446	
Number of activities per patient mobilised per ICU day	1.0 (0.4–1.8)	0.7 (0.3–3.0)	0.882
Number of episodes (total)	484	263	
Number of episodes per patient mobilised per ICU day	0.6 (0.3–1.0)	0.4 (0.2–1.3)	0.322
Percentage of patients who weight bear	47.0 %	29.3 %	<0.001^a^
Minutes per episode	105 (14–191)	157 (103–239)	<0.001^a^
Percentage of activities carried out on MV	12.9	35.9	<0.001^a^
Percentage of activities carried out with ETT	3.4	2.2	0.228
Percentage of episodes on MV	16.3	41.1	<0.001^a^
Percentage of episodes with ETT	2.1	2.7	.602
Day first mobilised (median)	2 (1–4)	3.5 (1–9)	<0.033^a^

*Abbreviations*: *ETT* endotracheal tube, *ICU* intensive care unit, *MV* mechanical ventilation

^a^Statistically significant

**Fig. 2:**
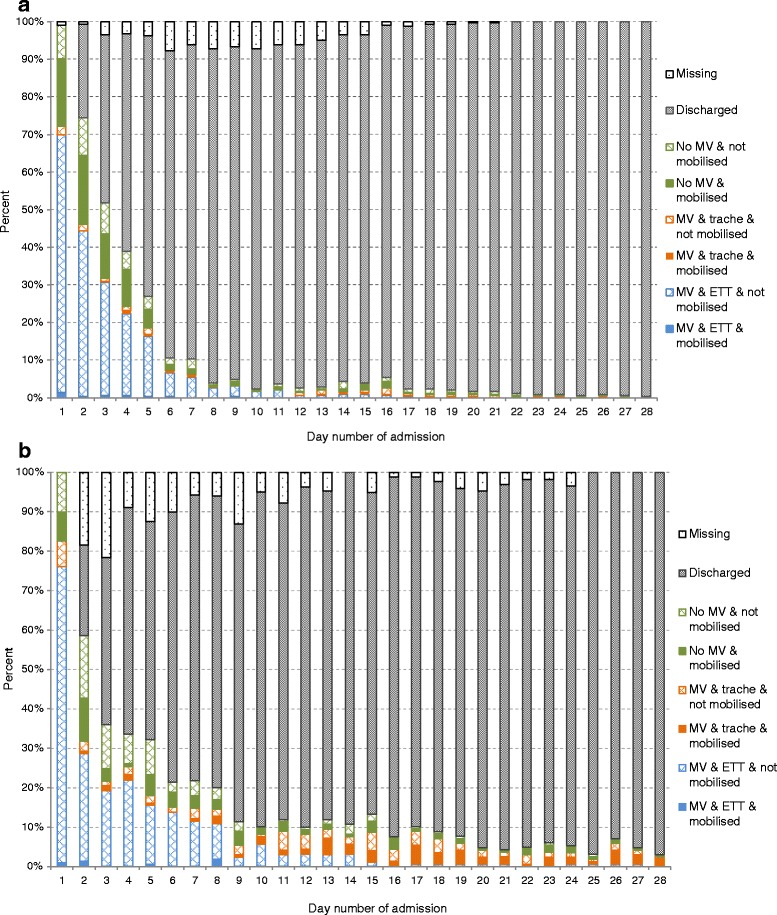
**a** Mobilisation status on each day of intensive care unit (ICU) admission for patients in the Australian cohort (n =347). **b** Mobilisation status on each day of ICU admission for patients in the Scottish cohort (n =167). *ETT* endotracheal tube, *MV* mechanical ventilation, *trache* tracheostomy

In total, more activities were conducted on mechanical ventilation in the Scottish cohort than in the Australian cohort (35.9 % vs 12.9 %; *p* < 0.001). Activities in the presence of an ETT were rare in both cohorts (Australia 3.4 %, Scotland 2.2 %; *p* = 0.228). Figure 2a and b displays mobilisation activities for Australia and Scotland for each day of patient admission as the percentages of patients with an ETT and mechanical ventilation, with a tracheostomy and mechanical ventilation or with no mechanical ventilation and the occurrence of mobilisation within each of these categories.

### Mobilisation and discharge destination

**Fig. 3:**
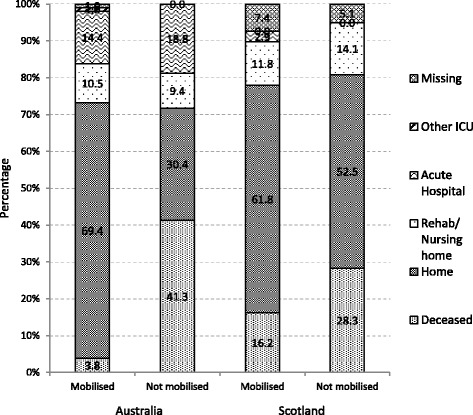
Discharge destination for Australian and Scottish patients who mobilised and those who did not mobilise. *ICU* intensive care unit

There was no difference in the mortality rate at hospital discharge between the Australian and Scottish arms of the study (18.7 % vs 23.4 %; *p* = 0.222). Figure 3 reports the hospital discharge destination of all patients who did and did not mobilise. Patients who were mobilised were discharged to home more often than those who did not mobilise in the Australian cohort (mobilised 69.4 % vs not mobilised 30.4 %; *p* < 0.001), but not in the Scottish cohort (mobilised 61.8 % vs not mobilised 52.5 % *p* = 0.237). Patients in the Australian cohort who were mobilised were less likely to die (3.8 % vs 41.3 %; *p* < 0.001), but this was not the case in the Scottish cohort (16.2 % vs 28.3 %; *p* = 0.069).

### Barriers to mobilisation

**Fig. 4:**
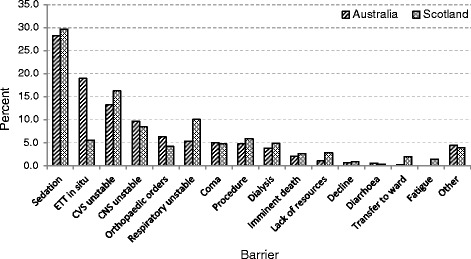
Reported barriers to mobilisation for each occasion of service in the Australian and Scottish cohorts. *CNS* central nervous system, *CVS* cardiovascular system, *ETT* endotracheal tube

Figure 4 summarises barriers to mobilisation for all patients in the Australian and Scottish cohorts, noting that patients could have more than one barrier reported on each occasion when mobilisation did not occur.

Sedation was the most commonly reported barrier in both the Australian and Scottish cohorts. Physiological instability in the CVS and CNS was also frequently reported as a barrier to mobilisation for both groups. The presence of an ETT featured more commonly as a barrier in the Australian cohort than in the Scottish cohort (18.1 % vs 5.4 %). However, respiratory failure was more commonly reported in the Scottish cohort (5.1 % vs 9.7 %).

## Discussion

This study has benchmarked and provided an international comparison of mobilisation practise in Australian and Scottish ICUs. Patients in the Australian cohort were more likely to mobilise and to mobilise sooner when in ICU than were patients in the Scottish cohort. However, patients in the Scottish cohort were more likely to mobilise whilst on mechanical ventilation, which may require more time and staff. With finite resources available to both cohorts, there appear to be different approaches to therapists’ practise in the two settings.

The percentage of mobilisation episodes with the presence of an ETT and mechanical ventilation was low in both the Australian (2.1 %) and Scottish (2.7 %) cohorts. However, mobilisation while on mechanical ventilation (which includes both via an ETT and via a tracheostomy) was significantly higher in the Scottish group than in the Australian group. Mobilising with ETTs has been reported in two previous studies. Bailey et al. [23] found 40.9 % of 103 study patients mobilised with an ETT, and Thomsen et al. [27] observed 60 % of 10 subjects mobilised with this therapy. One explanation for the discrepancy between previous studies conducted in the United States and the present study could be differences in timing of tracheostomy insertion. Patients in the US studies continued to have ETTs in situ at day 14 [23, 27, 30] in comparison with the present study, in which patients in both the Australian and Scottish cohorts often had an ETT in situ for less than 7 days. The lower number of days with an ETT gives less opportunity to mobilise with one in situ. However, mobilisation while on mechanical ventilation via a tracheostomy occurred on more than one-third of the possible occasions in the Scottish population. In recent studies, researchers have reported mobilisation on mechanical ventilation to be both safe and feasible [23, 26, 27, 30–32]; yet, this practise does not yet seem to be commonplace in the Australian cohort and only in patients with a tracheostomy in Scotland.

Analysis of mobilisation results was done after exclusion of patients admitted for cardiothoracic surgery in the Australian population. However, the differences between the cohorts in both admission diagnosis and mobilisation practises highlight the need for clear documentation of baseline practise before implementation of any change process. It also calls into question the generalisability of findings of studies carried out in ICUs of different countries and studies that have examined subgroups of the ICU patient population.

Sedation has been highlighted as a potential modifiable barrier to mobilisation [33]. In both the Australian and Scottish cohorts, sedation was the leading barrier to mobilisation. Although this is not surprising, it has not previously been reported on for all patient diagnoses in ICU for Australia and Scotland. This finding emphasises the need for a broad, multidisciplinary approach to be taken for future studies aimed at improving mobilisation rates.

Patient safety and stability are a common concern of clinicians when discussing barriers to mobilisation in the ICU [25, 34, 35]. The results of the present study are in keeping with these findings. Physiological instability of the CVS and CNS were reported as common barriers for both populations. In the present study, barriers were recorded at the patient’s bedside at the end of treatment by the physiotherapist. Responses therefore reflect individual clinician’s beliefs regarding why a specific patient did not mobilise. Although the responses may be subjective in nature, they are an accurate reflection of currently held beliefs on what was stopping patients from being mobilised. With the growing body of literature in this area showing consistently low adverse event rates with early mobilisation [23, 26, 27, 30–32], a question of what qualifies as physiological stability is raised. Furthermore, are the physiological assessments valid in predicting adverse events associated with mobilisation? In order to facilitate higher rates of mobilisation, boundaries of physiological stability must be challenged.

Differences in ICU setup may have contributed to some observed differences in barriers to mobilisation. The ICU size was smaller in Scotland, but the staffing ratios between Australia and Scotland were similar. A smaller unit size may mean that physiotherapy presence is not full-time in the unit but spread across wards and intensive care. This would explain the high reporting of ‘transferred to the ward’ and ‘procedure’ as barriers for Scottish ICUs, as patient discharge may have occurred before therapists had an opportunity to access the patient.

Although causation cannot be drawn from this data, a positive association was shown between patients who mobilised and those who were discharged to home in the Australian cohort. Future research should consider examining how patients are identified to mobilise and whether there are any modifiable factors that could increase the proportion of patients who are mobilised in the ICU.

## Conclusions

To our knowledge, this study is the first to document mobilisation rates and barriers to mobilisation at the bedside for all mechanically ventilated patients, inclusive of all diagnoses, in 19 ICUs across Australia and Scotland. Detailed baseline data on mobilisation activity and the barriers to it occurring are vital for informing future studies and accurately identifying the extent to which mobilisation influences patient-centred outcomes. The results of this study show that, though mobilisation was occurring in ICU, the proportion of patients mobilised with an ETT in situ was low.

Barriers to mobilisation are common and many. Sedation was consistently recorded as the leading barrier to mobilisation, with the perception of physiological instability being the second most important reason. Recommendations for future studies are to challenge what constitutes physiological stability in order to progress mobilisation as an intervention in ICU.

## Key messages

- Evaluation of mobilisation practises at the bedside shows large variations between units and countries.
- Mobilisation of patients with an ETT in situ is rare in both Australian and Scottish ICUs, despite recent evidence of its being a safe practise.
- Mobilisation of patients on mechanical ventilation was more commonly recorded for patients in Scottish ICUs than patients in Australian ICUs.
- Sedation is the most commonly reported barrier to mobilisation and should be considered when planning to improve mobilisation rates in future studies.

## Abbreviations

APACHE: Acute Physiology and Chronic Health Evaluation
CNS: central nervous system
CTx: cardiothoracic surgery
CVS: cardiovascular system
ETT: endotracheal tube
ICU: intensive care unit
IQR: interquartile range
LOS: length of stay
MV: mechanical ventilation
NHS: National Health Service
SD: standard deviation
